# Emerging Therapies for Stage III Non-Small Cell Lung Cancer: Stereotactic Body Radiation Therapy and Immunotherapy

**DOI:** 10.3389/fonc.2017.00197

**Published:** 2017-09-04

**Authors:** Sameera S. Kumar, Kristin A. Higgins, Ronald C. McGarry

**Affiliations:** ^1^Department of Radiation Medicine, University of Kentucky, Lexington, KY, United States; ^2^Department of Radiation Oncology, Winship Cancer Institute of Emory University, The Emory Clinic, Atlanta, GA, United States

**Keywords:** stereotactic body radiation therapy, immunotherapy, radiation therapy, non-small cell lung cancer, stage III

## Abstract

The current standard of care for locally advanced non-small cell lung cancer (NSCLC) includes radiation, chemotherapy, and surgery in certain individualized cases. In unresectable NSCLC, chemoradiation has been the standard of care for the past three decades. Local and distant failure remains high in this group of patients, so dose escalation has been studied in both single institution and national clinical trials. Though initial studies showed a benefit to dose escalation, phase III studies examining dose escalation using standard fractionation or hyperfractionation have failed to show a benefit. Over the last 17 years, stereotactic body radiation therapy (SBRT) has shown a high degree of safety and local control for stage I lung cancers and other localized malignancies. More recently, phase I/II studies using SBRT for dose escalation after conventional chemoradiation in locally advanced NSCLC have been promising with good apparent safety. Immunotherapy also offers opportunities to address distant disease and preclinical data suggest immunotherapy in tandem with SBRT may be a rational way to induce an “abscopal effect” although there are little clinical data as yet. By building on the proven concept of conventional chemoradiation for patients with locally advanced NSCLC with a subsequent radiation dose intensification to residual disease with SBRT concurrent with immunotherapy, we hope address the issues of metastatic and local failures. This “quadmodality” approach is still in its infancy but appears to be a safe and rational approach to the improving the outcome of NSCLC therapy.

## Chemoradiation in Stage III Non-Small Cell Lung Cancer (NSCLC)

One hundred years ago, lung cancer was a rare malignancy (1). Lung cancer today is the leading cause of cancer death in the United States, with over 158,000 estimated deaths in 2016 (2). Forty percent of these patients present with locally advanced disease (3). Approximately 80–90% of newly diagnosed lung cancers are classified as NSCLC, primarily consisting of adenocarcinoma, squamous cell carcinoma, or large cell carcinoma histologies. Historically, surgery has been the gold standard for newly diagnosed NSCLC with early-stage resectable disease, resulting in 5-year overall survival rates (OS) of 50–70%. However, for patients with more locally advanced NSCLC, 5-year OS after treatment with definitive radiation therapy and concurrent chemotherapy remains modest, at approximately 15–20% (4). Prior to the advent of cytotoxic chemotherapy, lung cancer at all stages was treated surgically or by radiation alone (5, 6). TNM staging was introduced in 1974 and it helped shape the way lung cancer was managed. Stage III lung cancer, though heterogeneous in its classification, includes non-metastatic but locally advanced disease with involvement of N1–N3 nodal stations and/or T3 and T4 primaries. Presently, stage III lung cancer is managed with a combination of chemotherapy, radiation therapy, and sometimes surgery but the two major challenges in improving outcomes of the treatment of this disease remain local control and metastatic spread.

## Chemoradiotherapy (CRT) Dose Issues

Multiple studies have examined the issue of the optimal dose of radiotherapy in NSCLC but are complicated by the heterogeneity of the disease itself in terms of size and location of the primary tumor, number and size of involved lymph nodes, and the patient’s comorbidities, all of which limit the treatment tolerability and risks. Delivery of tumoricidal doses to the primary tumor and involved lymph nodes is balanced by treatment-related toxicities, namely esophagitis, pneumonitis, and cardiac injury.

An early dose-finding study by the Radiation Therapy Oncology Group (RTOG) 7301 study was conducted from 1973 to 1978 and studied four different doses and schedules: 40 Gy split course, 40 Gy continuously, 50 Gy, and 60 Gy. All doses were given in 2 Gy fractions. The optimal dose was determined to be 60 Gy (7).

Further improvements in survival were sought by the incorporation of chemotherapy. The Cancer and Leukemia Group B 8433 study solidified chemotherapy’s importance in the treatment of locally advanced lung cancer. In this phase III study, 155 patients with stage III NSCLC were randomized to receive 60 Gy in 30 fractions or induction chemotherapy consisting of two cycles of cisplatin and vinblastine followed by 60 Gy in 30 fractions. Both median OS (13.8 versus 9.7 months) and 3-year OS were improved in the CRT arm (23 versus 11%) (8). Likewise, a European Organisation for Research and Treatment of Cancer study showed a benefit to concurrent CRT by randomizing patients to split-course radiotherapy alone to a dose of 55 Gy, split-course radiotherapy plus low-dose daily cisplatin, and split-course radiotherapy plus higher dose weekly cisplatin. The most salient differences were seen between concurrent daily CRT and radiation alone with the 3-year OS for CRT being 16 versus 2% for radiotherapy alone. This difference was thought to be due to an improvement in local control, as the 2-year local control in the daily CRT arm was 31 versus 19% in the radiotherapy alone arm (9).

Only one phase III trial has compared the traditional standard of 60 Gy to a modestly escalated dose regimen of 74 Gy. Based on the results of RTOG 0117 suggesting that 74 Gy represented a maximum tolerated dose of CRT for most patients, RTOG 0617 compared 60 versus 74 Gy both combined with weekly carboplatin and paclitaxel. In this four-arm study, a second randomization of cetuximab versus observation was also studied. Unfortunately an interim analysis showed that the 74 Gy arm had increased risk of death, with a median survival of 20 months for patients receiving 74 Gy versus 29 months for patients receiving 60 Gy, leading to early termination of the study (10). There was no benefit to local control. Of note, the 60 Gy arm had the highest median survival demonstrated within a phase III trial for this patient population. On multivariate analysis, increased dose to the heart, represented as heart V5 and V30 (the percent volume receiving ≥5 and ≥30 Gy, respectively), maximum esophagitis grade, planning target volume, and radiation dose (74 Gy) were all shown to negatively impact overall survival. There were no statistically significant differences in ≥grade 3 toxic effects between the groups; however, heart-specific toxicities were not assessed in this trial. Ultimately this underlines the difficulty of dose escalation with conventional radiation therapy fractionation techniques in the general population of patients with stage III NSCLC, opening the door for new strategies to improve outcomes for locally advanced disease. Often the argument is put forth that surgery is the ultimate form of local control and indeed 5-year local control rates for locally advanced NSCLC after CRT have been reported as low as 15%, but at least some of this is possibly biased by the selection of more resectable patients receiving surgery (11). Improving local control of the primary lesion in NSCLC does influence overall survival, as demonstrated by a meta-analysis of concurrent CRT versus sequential chemotherapy and radiation (12). Thus, if radiation techniques could be optimized and local control improved, one could expect to see improvement in long-term patient survival.

## Individualized CRT

Since most dose-escalation studies have produced problematic results in relatively unselected patients, can escalated radiation doses safely be delivered to patients by adaptive radiotherapy either during or after conventional radiotherapy? Additionally, in an era of intense research into molecular markers and innovative systemic therapies, how can combination strategies best be utilized to improve both local control and risk of metastasis?

Radiation Therapy Oncology Group 9311 was an early multi-center dose-escalation trial of 179 patients which used radiotherapy alone (13). The treatment was individualized based on the volume of lung receiving 20 Gy or more (V20). Those with a V20 less than 25% were dose-escalated to 90.3 Gy. Those with a V20 of 25–36% were dose-escalated to 83.8 Gy. Both schemes were performed at 2.15 Gy per fraction. Two treatment-related deaths occurred in the 90.3 arm and this dose was labeled as too toxic. Elective nodal coverage was not allowed, but still the isolated nodal failure rate was less than 10%. For the group with a V20 less than 25%, 83.8 Gy was found to be safe and for the group with a V20 of 25–36% 77.4 Gy was found to be safe.

More recently Kong et al. reported results of a phase II study of mid-treatment positron emission tomography-computed tomography (PET/CT) adapted radiotherapy with concurrent chemotherapy (14). Briefly, in this study, 43 patients with unresectable stage II–III NSCLC received radiotherapy with doses individualized for an allowable mean lung boost dose of up to 20 Gy which would produce a risk of pneumonitis up to 17.5%. Radiation was delivered in 30 fractions with all patients receiving 2.1–2.85 Gy/fraction for the initial dose up to approximately 50 Gy EQD2 with the adaptive phase of the treatment of 2.85–5.0 Gy/fraction for a total radiation dose of up to 86 Gy in an attempt to deliver >100 Gy BED_10_. Weekly carboplatin and paclitaxel were given concurrently. After a median follow-up of 47 months, the 2-year infield and overall local regional tumor controls were 82 and 62%, respectively; and median OS was 25%. Overall these results are consistent with most other stage III studies. This promising strategy of mid-treatment PET with dose escalation is currently being evaluated in the RTOG 1106 randomized trial, which recently completed accrual. Though local control has improved with these trials, metastatic disease still remains an important site of failure.

Stereotactic body radiation therapy (SBRT) has changed the standard of care for early-stage lung cancer, and data are emerging showing applicability to the stage III NSCLC population. The evidence for a role of SBRT in the stage III lung cancer population is summarized within this review.

## Biologically Effective Dose (BED) and SBRT

The success of SBRT treatments in early-stage NSCLC likely reflects the radiobiologic properties of high radiation doses. Higher radiation doses result in exponential increases in cell kill, and may also have an ablative effect on tumor vascularity and stroma (15, 16). A method of dose modeling based on the linear quadratic model of cell killing, referred to as the BED, takes into account the radiation dose per fraction and the inherent radiation response of a particular tissue (17). As derived from linear quadratic curves, mathematically two different dose and fractionation schemes can be compared theoretically for tumor control probability. An important assumption of this model is referred to as the α/β ratio, simplistically thought of as the ratio of cell killing based on single hit and multi-hit kinetics that leads to local control of a cancer mass (primarily from cell culture experiments, animal data and clinical observation). Nevertheless, tumor control probabilities are more complicated than a simple mathematical statement since tissues are complicated structures with underlying vasculature, stroma, and tumor cells, all of which interact (18). Many of the α/β assumptions are, therefore, also based on long clinical observation of tumor control and normal tissue toxicities. The BED equation can be expressed as BED = *nd*(1 + *d*/α/β) where *n* = the number of fractions, *d* = the dose/fraction, and α/β = alpha-beta ratio. Often early-reacting tissues/tumor cells are considered to have an α/β of approximately 10 whereas late reacting tissues are assigned an α/β of approximately 3. Based on these assumptions, Martel et al. constructed a mathematical model which predicted that in NSCLC a dose 84 Gy must be achieved for a local progression-free survival (PFS) of greater than 30 months (19). A retrospective study found that the doses of at least 70 Gy at 1.8–2 Gy per fraction provided better local control and survival for tumors less than 100 cc (20). Using 2 Gy fractions, a dose of 70 Gy has a BED of 84 Gy.

Based on the success of Gamma Knife treatment of brain lesions, extremely hypofractionated extracranial stereotactic radiotherapy programs began in the 1990s and are commonly known as SBRT or stereotactic ablative radiation therapy. SBRT treatments, because of the high dose per fraction, are able to achieve a much higher BED to localized volumes than conventional radiation delivered at 2 Gy/fraction. Multiple studies have demonstrated that a higher BED is correlated with improved local control and survival (21–24). Onishi et al. have shown that in early-stage lung cancer, superior local control and survival are achieved with treatment regimens that reach a BED of 100 Gy or greater (21). Specifically in lung cancer, SBRT delivers a high dose per fraction, with robust immobilization that minimizes intra-fraction motion and tumor-related internal motion, allowing for overall reduction in size of treatment volumes and overall treatment time.

In the seminal clinical reports by Blomgren and Lax, the philosophy and treatment parameters for the hypofractionated highly conformal treatment of localized disease that we use today were elucidated (25). In an *ad hoc* manner, they treated a number of different sites of localized disease most notably early-stage lung cancers settling on a dose of 60 Gy in three fractions of 20 Gy each with excellent local control and minimal toxicity. Their studies defined the parameters required for safe and precise delivery that we utilize in SBRT delivery today. Presciently, they speculated that “this new technique may also be used for delivering boost doses with a high precision after conventional radiation therapy” (26).

## SBRT Clinical Trials in Early-Stage Lung Cancer

In an effort to better define SBRT doses for localized disease, Timmerman et al. performed a phase I-II dose-escalation study for SBRT to the primary tumor in patients with stage I NSCLC using the concept derived from Swedish studies (27). Inhomogeneity corrections to correct for lung density were not performed. Separate cohorts of patients were followed with the dose-escalation ending at 60 Gy in 20 Gy fractions with no dose-limiting toxicity. Termination of the dose escalation for these smaller tumors was based on modeling of cell kill. For larger tumors (up to 7.0 cm) a dose-limiting toxicity (pneumonitis) was reached at 72 Gy in 24 Gy fractions. This experience laid the groundwork for further national clinical trials evaluating SBRT as a therapy for medically inoperable early-stage NSCLC, and ultimately changed the standard of care for these patients. Currently, SBRT is defined as 1–5 treatments of high-dose radiation delivered to tumors, typically measuring up to 7 cm, with registration of the patient’s anatomy to a 3-D coordinate system either physical or within the planning system. SBRT is considered an ablative treatment intended to disrupt cellular clonogenicity, and lead to cell death. Robust immobilization, control of internal organ and tumor motion, sharp dose gradients, and high dose per fraction (≥600 cGy) for five or fewer fractions have been considered to define SBRT.

The first North American prospective cooperative group clinical trial evaluating SBRT, RTOG 0236 began accrual in 2004 and only allowed “peripherally located” tumors as defined by being outside 2 cm of the proximal bronchial tree or mediastinum (commonly referred to as the “no fly” zone). This study accrued 59 patients, treated with 18 Gy × 3 (total 54 Gy with heterogeneity corrections) to the primary tumor, and demonstrated 3-year local control (involved tumor and primary lobe) of 91% for patients with T1-2, N0 medically inoperable lung cancer (28). Three-year local–regional control was 87%, and distant failure rate was 22%. Overall survival was 56%. Results from longer follow-up have shown higher rates of local failure, primarily due to intralobar recurrences, with 5-year local recurrence rates of 20% (29). Importantly, these clinical outcomes are far better than historical studies treating medically inoperable early-stage lung cancer with conventionally fractionated radiation (2 Gy/fraction), with dismal local control of the primary tumor of 50% or less (30). Grade 3 and higher adverse events occurred in approximately 15% of patients enrolled in RTOG 0236.

For centrally located tumors, RTOG 0813 was a phase I-II study for T1-2, N0 medically inoperable lung cancer 5 cm or less in size, centrally located within or touching the 2 cm bronchial tree “no fly” zone. The primary endpoint was to establish the optimal SBRT dose for centrally located tumors. With dose cohorts of 10 Gy × 5, 10.5 Gy × 5, 11 Gy × 5, 11.5 Gy × 5, and 12 Gy × 5, it was found that the highest dose cohort had a 7% probability of a dose-limiting toxicity (31). RTOG 0915 was a randomized phase II study designed to test 34 Gy × 1 versus 12 Gy × 4 for non-centrally located tumors, with a primary endpoint of determination of the regimen with the lowest rates of protocol specified adverse events at 1 year. One year adverse events were 10% for the 34 Gy arm, and 13% for the 48 Gy arm (32).

It thus appears that there are multiple hypofractionated schemes that are acceptable using SBRT techniques to achieve high degrees of local control but they all have one thing in common: BED > 100.

## SBRT Toxicity

Though grade 3–5 toxicities with SBRT are overall low, Timmerman et al. retrospectively found in the initial single institution phase II study that 20–22 Gy × 3 was overly toxic for tumor in a central location, defined as within 2 cm from the proximal bronchial tree. In this phase II study, 2-year freedom from severe toxicity was 83% in patients with peripheral tumors and 54% for patients with central tumors (33). A separate single institution study recently showed a 3.7% fatal toxicity rate for SBRT with central tumors, with tumors abutting the proximal bronchial tree having significantly more grade 3+ adverse events (31 versus 7%) (34).

This suggests that tumor location with regards to the potential for late toxicity attributable to SBRT may be important as described above, but the RTOG 0813 SBRT dose-escalation study shows that central tumors may be safely treated to significant SBRT doses (31). As data and experience accumulates, dose-limiting organs within the hilum and mediastinum are becoming better defined and with care, SBRT can be utilized to treat “central” tumors safely.

Nonetheless, a large body of literature is accumulating confirming that SBRT treatment is well tolerated and safe in patients who are medically inoperable with early-stage lung cancer and produces excellent results. The question of applying SBRT to a stage III population with centrally located mediastinal lymph nodes as well as primary tumors remains pertinent. The studies summarized below describe the experience of SBRT in the locally advanced, stage III patient population.

## Dose-Escalated Hypofractionated Radiation (SBRT) in Stage III NSCLC

Investigators at the University of Kentucky completed a prospective study evaluating the feasibility of conventional CRT followed by a SBRT boost to the primary tumor as a method to dose escalate in patients with residual disease following CRT (35). In this study, patients with stage IIIA and IIIB NSCLC received CRT (median dose of 59.4 Gy) followed by a whole body fluorodeoxyglucose-positron emission tomography (FDG-PET) scan 1 month after treatment. Eighty-nine percent of patients received concurrent, platinum-based chemotherapy during CRT. Patients were eligible for SBRT if they had evidence of residual disease at the primary tumor location that was ≤5 cm in greatest dimension. Patients with progressive metastatic disease, contralateral lung disease or residual disease in the hilum or mediastinum were not eligible (defined as SUV ≥ 2). SBRT doses were 6.5 × 3 for centrally located primary tumors, and 10 Gy × 2 for non-central tumors. With these dose schemas, the cumulative BED10 to the primary tumor was 110 Gy for non-central tumors and 102 Gy for centrally located tumors. Sixty-two patients were screened, and 37 patients were ultimately eligible and enrolled. Approximately 31% of patients screened had new metastatic disease and an additional 31% had persistent nodal disease on post-treatment FDG-PET. The primary endpoint of this study was to assess the proportion of patients who developed ≥grade 3 radiation pneumonitis, according to the RTOG acute and late radiation morbidity scoring criteria. Overall, 11.4% of patients experienced radiation pneumonitis consistent with rates found in most studies of conventional CRT suggesting no increase risk with the SBRT boost. Two patients developed fatal pulmonary hemorrhage felt to be possibly related to treatment but careful analysis showed that these cases were more likely to have been related to squamous cell cavitary recurrences involving the hilum (36). Statistically there were no differences dosimetrically between patients who developed a fatal hemorrhage from those who did not. Local recurrence remained the most significant predictor. The central structures including the bronchial walls, pulmonary arteries, and aorta were contoured and the individual doses delivered to these structures were compared as well as the location of the PTV to the hilum. This small series of patients suggested that it is prudent to restrict the maximum radiation dose to the pulmonary artery to less than 185 Gy cumulative BED_3_, and to less than 120 Gy BED_3_ for the 5 cc volume; as well as limiting the maximum dose to the bronchial wall to less than 175 Gy BED_3_. The equivalent dose on a per fraction basis would be equivalent to limiting each of these structures to less than 700 cGy per fraction times 3, or 900 cGy per fraction times 2 for the boost, assuming that the patient has previously received between 60 and 66 Gy using standard fractionation. The most recently reported long-term follow-up of this study shows a crude local control rate of 78%. Median overall survival was 25 months. There were no significant late toxicities seen within the study population (37).

Second, a recent phase I study by Higgins et al. (in press) evaluated the optimal SBRT dose after 44 Gy CRT. Inclusion criteria included stage IIIA or IIIB NSCLC, with a primary tumor of 8 cm or less and no N1 or N2 nodal station >5 cm in maximum dimension. This multi-institution phase I study enrolled 15 patients, and dose-escalated a SBRT boost according to the following dose cohorts: 9 Gy × 2, 10 Gy × 2, 6 Gy × 5, 7 Gy × 5. Patients received 44 Gy with weekly carboplatin and paclitaxel, and then underwent a second computed tomography (CT) simulation after 40 Gy was delivered. The SBRT boost was then planned to encompass all residual primary and nodal disease as seen on the planning CT simulation. This volume was then dose-escalated according to the dose assignment of the patient. The maximum tolerated dose was determined to be 6 Gy × 5. There was one treatment-related grade 5 toxicity at this dose level, and 10 Gy × 2 is felt to be the most optimal SBRT boost dose, as no grade 3 or higher toxicities were seen in patients treated within the dose cohort. For all patients, actuarial local regional control at 3 years was 59%, and 3-year overall survival was 39% (38).

In an additional phase I study by Hepel et al., 12 patients with stage III NSCLC who had a primary tumor volume <120 cc (approximately 6.0 cm) and nodal disease volumes <60 cc received CRT to a dose of 50.4 Gy in 28 fractions (39). The study used a dose-escalation design to identify the maximum tolerated dose. SBRT dose was escalated from 16 Gy in two fractions to 28 Gy in two fractions in 2 Gy/fraction increments, resulting in four potential dose cohorts. The endpoint was dose-limiting toxicity occurring within 4 weeks of SBRT. A standard phase I cohort design was used. SBRT cohort doses started at 800 cGy × 2 fractions and escalated by 200 cGy/fraction to a final dose of 1,400 cGy × 2 for a total SBRT boost of 28 Gy. No early grade 3–5 toxicities were noted and at a median follow-up of 16 months, 1 year local–regional control was 78% with 100% at ≥24 Gy. Overall survival at one year was 67%. One late fatal pulmonary hemorrhage was noted and it was determined that the patient’s 4 cc proximal bronchial-vascular tree dose was substantially higher than all patients reported at 30.2 Gy for the SBRT boost and 73.5 Gy for the total treatment. A total BED computation was not available to assess all patient doses.

It is clear from these studies and RTOG 0813, contouring of at risk structures and applied dose constraints (see above estimates) particularly for the pulmonary vasculature need to be respected in the treatment plan.

## Immunotherapy in NSCLC

The use of immunotherapy in NSCLC is rapidly burgeoning. Early vaccine trials and trials with interferon therapy for those who were suffering from NSCLC have been largely negative and led to the hypothesis that NSCLC was believed to be largely non-immunogenic. Clearly, the immune response must be tightly controlled to prevent rampant autoimmunity. Multiple mechanisms to regulate immune responses have been shown to exist including innate tolerance to self-antigens, a network of both B and T suppressor cells and more recently elucidation of molecular regulatory mechanisms including checkpoint inhibitors. Immune checkpoint inhibitors have shown some promise in modulating the tumor microenvironment so that evasion of the immune system is more difficult.

Surveillance and destruction of tumor cells is postulated to be effected by the immune system and the vanguard of early tumor control may be the natural killer cell although its full role is yet to be elucidated. Once a tumor is established, control may be mediated by activated T-lymphocytes including CD4+ and CD8+ cells. The CTLA-4 and programmed death ligand 1 (PD-1) pathways are two T-cell inhibitory pathways that may modulate immune responses to lung antigens in the presence of an increasing burden of malignant cells possibly in an effort to prevent damage to host normal tissues. Inadvertently this may result in suppression of the immune system favoring tumor cell survival and growth. A CTLA-4 monoclonal antibody which is currently in use is ipilimumab, currently indicated in the treatment of melanoma. The PD-1 receptor ligands include PD-L1 and PD-L2. Nivolumab and pembrolizumab are two PD-1 inhibitors which have been FDA approved for clinical use in lung cancer.

Several seminal trials suggested the utility of blocking the PD inhibitory pathway by monoclonal antibodies to harness the immune system in control of NSCLC. The Checkmate 057 phase III clinical trial randomized 582 patients with non-squamous metastatic NSCLC who had progressed during or after platinum-based chemotherapy to salvage docetaxel chemotherapy or nivolumab. Median OS was longer in the nivolumab group (12.2 versus 9.4 months). Patients with even <10%, but greater than 1% PD-L1 expression showed a benefit with nivolumab over docetaxel (40). A second study, Checkmate 017, studied 272 patients with metastatic squamous cell NSCLC who progressed through platinum-based first-line chemotherapy. Those who received nivolumab had a median OS of 9.2 months versus those who received docetaxel, with a median OS of only 6.0 months (41). The use of nivolumab as a first-line agent was explored in the phase III Checkmate 026 trial in which 541 patients with previously untreated metastatic NSCLC with at least 1% PD-L1 expression were randomized to nivolumab or standard-of-care platinum doublet chemotherapy. Both PFS and OS were not significantly different between the two arms (42).

The KEYNOTE-010 trial enrolled over 1,000 patients with previously treated advanced NSCLC with at least 1% PD-L1 expression. They were randomized to two different doses of pembrolizumab or docetaxel. Median OS was 10.4 months with 2 mg/kg of pembrolizumab, 12.7 months with 10 mg/kg of pembrolizumab, and 8.5 months with docetaxel, which was statistically significant. An even greater survival benefit was seen in those with >50% tumor PD-L1 expression: 14.9 months with 2 mg/kg of pembrolizumab, 17.3 months with 10 mg/kg of pembrolizumab, and 8.2 months with docetaxel, which was also statistically significant (43). As a first-line therapy, the phase III KEYNOTE 024 trial explored the use of pembrolizumab in advanced NSCLC with at least 50% PD-L1 staining versus cytotoxic chemotherapy, which was up to the discretion of the treating physician. Only 30% of the patients had the required 50% or greater PD-L1 staining tumors. In those patients, pembrolizumab was seen to significantly increase the 6-month OS (80.2 versus 72.4%) (44).

PD-L1 reactive monoclonal antibodies are currently being explored in NSCLC. Atezolizumab is one such IgG1 agonist to PD-L1. In the OAK trial, 1,225 patients with advanced NSCLC were randomized to salvage chemotherapy with docetaxel or atezolizumab. Greater OS was seen with atezolizumab regardless of PD-L1 expression (13.8 versus 9.6 months) (45).

To date, there are only limited data from phase III trials regarding immunotherapy for stage III NSCLC. The phase III START trial enrolled 1,514 patients with stage III NSCLC who had received CRT and had not progressed within 1–3 months. Patients were randomized to either placebo or tecemotide, an anti-MUC-1 immunotherapy designed to stimulate a T-cell response against the MUC-1 protein. There was no OS difference between the placebo group and the tecemotide group, except in a subgroup receiving concurrent CRT. In this case, the tecemotide group did have an improved OS (46), suggesting a possible synergistic interaction between the radiation and the drug. Belagenpumatucel-L is a tumor vaccine of four allogeneic NSCLC cell lines. In a phase III trial, 270 stage III or IV patients who were treated with platinum-based chemotherapy and who had not progressed were randomized to receive placebo or belagenpumatucel-L. There were no differences in OS or PFS between the two arms (47). A killed *Mycobacterium vaccae* named SRL172 was the subject of a phase III clinical trial published in 2004. A total of 419 patients were treated with 6 cycles of mitomycin, vinblastine and cisplatin or carboplatin with or without monthly administration of SRL172. There were no differences in overall survival, but patients in the SRL172 arm reported better quality of life (48). A meta-analysis of 20 trials by Zhou et al. found an OS benefit to immune checkpoint inhibitors and therapeutic vaccine (49).

## Rationale for the Use of Immunotherapy with Radiotherapy

Immunogenic cell death is a postulated mechanism of radiation injury. Classically it is thought that the immune system must recognize either foreign (e.g., viruses) or mutated antigens on tumor cells to initiate an immunostimulatory response. Thus far, no simple antigen has been identified since in many ways, cancer cells are “self.” Roszik et al. found a significant relationship between the predicted tumor mutation load and clinical benefit from ipilimumab, T-cell therapy, and pembrolizumab suggesting mutated proteins or DNA-protein complexes may be immunostimulatory (50). Unlike conventional apoptosis, when due to an immunogenic cell death apoptosis causes a release of molecules which may lead to an inflammatory or augmented immune response (51, 52). Damaged cells produce damage-associated molecular patterns which lead to uptake and subsequent presentation of tumor antigen by dendritic cells. Radiation has been shown to release or upregulate immune and tumor-related molecules such as major histocompatibility complex, tumor markers, adhesion molecules, cytokines, and many others (53). Single doses of 15–25 Gy induced strong T-cell responses, but these immune responses were dampened by the use of fractionated radiation or chemotherapy (54). Unfortunately, since lymphocytes are so radiosensitive, only a low integral dose is needed to kill any surrounding tumor lymphocytes. There is some evidence that ablative radiation fraction sizes (at least 6 Gy) or high linear energy transfer radiation causes increased release of immunogenic antigens. Mouse studies have shown evidence of the abscopal effect after use of large fractions (55, 56). A paper by Lugade et al. looked at 15 Gy in a single fraction versus 15 Gy in 5 fractions of 3 Gy in in a mouse melanoma model. They found that both fraction sizes lead to tumor-infiltrating lymphocytes that were capable of lysing tumor cell targets, but that the larger fraction size produced better results (57). A strong antitumor immunogenic response was observed in mouse models after being treated with a carbon ion beam. This resulted in fewer contralateral squamous cell tumors, which is thought to be due to an immune-mediated abscopal effect (58). Strictly defined, the abscopal effect is the resulting shrinkage or disappearance of metastatic deposits following treatment of the primary tumor mass. Clinically the abscopal effect is rarely seen, with fewer than 50 documented cases in the literature (59). Barid et al. propose that this is because while radiotherapy provides available antigen, it does not provide the necessary co-stimulation of T cells or cytokine release (60). Thus, this presents an opportunity for the combined use of radiotherapy and immunotherapy.

Both laboratory and clinical evidence exist regarding the advantage of combined radiotherapy and immunotherapy. In a murine model of metastasis, squamous cell carcinoma cell lines were inoculated into the mouse thigh typically requiring ≥10^6^ tumor cells to ensure tumor growth. Most of these cells die and release tumor lysis products which may bias treatment results. Mice were treated with a single 6 Gy dose of carbon ions and 36 h later treated with α-galactosylceramide-pulsed dendritic cells. Compared to the untreated control mice, these mice developed significantly fewer pulmonary metastases (61). Intravenous administration of isolated dendritic cells with either carbon beam therapy or photon beam therapy was compared in a murine model. Both types of irradiation produced an antimetastatic effect, but carbon ions did so at a lower BED (62). Sharabi et al. examined the effect of SBRT in murine melanoma or breast cancer and found that the effect of radiation was enhanced in the presence of a PD-1 inhibitor or regulatory T-cell depletion (63). Some studies suggest that an immune-mediated abscopal effect is increased with fractionated radiotherapy using large fractions in addition to a CTLA-4 inhibitor as opposed to single-dose radiotherapy (64). Indeed, further mouse studies confirmed that fractionation using “medium-sized doses” (7.5 Gy per fraction) provided both low numbers of regulatory T-cells and the best control of the tumor (65).

Clinical studies also show encouraging results of the use of combined radiation and immunotherapy. Abscopal effects in humans after SBRT with or without immunotherapy have been reported in both renal cell carcinoma and melanoma (66–68). The KEYNOTE-001 study predated the KEYNOTE-010 study. KEYNOTE-001 was a phase I clinical trial which enrolled 495 patients with advanced NSCLC. They were treated with pembrolizumab at doses of 10 mg/kg every 3 weeks or 10 mg/kg every 2 weeks. The objective response rate was found to be 19.4% and OS was 12 months. In patients with at least 50% expression of PD-L1 median overall survival was not reached. It was deemed to have an acceptable side effect profile and the most common side effects included fatigue, itching, and decreased appetite (69). An analysis of the trial was done and showed that in 97 patients who had prior radiation PFS and overall survival were significantly longer, especially for those who received extracranial radiotherapy (70). In the PACIFIC study, a phase III study for stage III unresectable lung cancer, patients in the experimental arm received chemoradiation followed by durvalumab for 12 months. In a preliminary report, Astra Zeneca suggests an improvement in PFS in the immunotherapy arm was seen, however, these data have yet to be presented (71). Currently, there are several ongoing clinical trials investigating the use of immunotherapy with radiotherapy. These trials include agents such as cancer vaccines, CTLA-4 inhibitors, PD-1 inhibitors, and PD-L1 inhibitors (Table 1). This table was generated by searching the ClinicalTrials.gov database with search terms such as “radiation,” “chemoradiation,” “thoracic RT,” and several variations. The results were then manually filtered for the inclusion of Immunotherapy.

**Table 1:** Active clinical trials involving the use of both radiotherapy and immunotherapy such as cancer vaccines, CTLA-4 inhibitors, PD-1 inhibitors, and PD-L1 inhibitors in Stage III non-small cell lung cancer (NSCLC).

NCT Number	Title	Recruitment	Study results	Phase	Enrollment
NCT02987998	Neoadjuvant chemoradiation plus pembrolizumab followed by consolidation pembrolizumab in NSCLC	Recruiting	No results available	Phase 1	20
NCT02662634	A safety and feasibility study of AGS-003-LNG for the treatment of stage 3 NSCLC	Recruiting	No results available	Phase 2	20
NCT02434081	NIvolumab consolidation with standard first-line chemotherapy and radiotherapy in locally advanced stage IIIA/B non-small cell lung carcinoma	Recruiting	No results available	Phase 2	43
NCT02318771	Radiation therapy and MK-3475 for patients with recurrent/metastatic head and neck cancer, renal cell cancer, melanoma, and lung cancer	Recruiting	No results available	Phase 1	40
NCT02621398	Pembrolizumab, paclitaxel, carboplatin, and radiation therapy in treating patients with stage II-IIIB NSCLC	Recruiting	No results available	Phase 1	30
NCT02768558	Cisplatin and etoposide plus radiation followed By nivolumab/placebo for locally advanced NSCLC	Recruiting	No results available	Phase 3	660
NCT02125461	A global study to assess the effects of MEDI4736 following concurrent chemoradiation in patients with stage III unresectable NSCLC (PACIFIC)	Ongoing, but not recruiting	Active, not recruiting	Phase 3	713

## Summary

Treatment of locally advanced lung cancer has not made great strides since the 1990s when cytotoxic chemotherapy was combined with radiation. The two major stumbling blocks to improvements in survival of these patients are local control and distant metastasis. It is clear that SBRT for stage I NSCLC is one of the most important treatment advancements in decades with excellent outcomes of high local tumor control and survival with low toxicity.

Cytotoxic chemotherapy remains an important modality in more advanced disease but has reached a point where major improvements are unlikely and despite systemic therapy, metastatic disease is a prominent cause of death in locally advanced NSCLC patients.

We need more innovative approaches to management of this disease. Evidence is accumulating that dose escalation of radiotherapy improves local control of much of the microscopic and gross disease in the chest. Since dose escalation by conventional radiation delivery has been compromised by toxicity, the careful delivery of hypofractionated radiation therapy (SBRT) to the sites of gross disease should improve local control by ablating any residual viable cancer cells. The initial studies of SBRT boost while small, show this approach is safe and feasible, but the impact of this approach on survival in the management of stage II-III awaits larger studies.

The sequencing and combination of this “quadmodality” approach is still being explored. In the Phase I/II studies described above, concurrent chemoradiation to a dose of 44–60 Gy was used which was followed by an SBRT boost. The trials showed favorable toxicity profiles using this approach. Fractionated chemoradiation promotes immunotolerence through the killing of lymphocytes by the chemotherapy and radiation therapy, but SBRT has been shown to induce strong T-cell responses. Thus ideally the patient would undergo concurrent chemoradiation to a dose of 44–60 Gy, have an approximately 2-week break to allow for SBRT treatment planning and recovering from leukopenia, then get an SBRT boost. In order to capitalize on the immunostimulatory effects of the SBRT, the immunotherapy should be administered soon (within 1 week) of the SBRT boost. Cranial stereotactic radiosurgery with concurrent immunotherapy appears to be well tolerated, but data on lung SBRT and concurrent immunotherapy is still developing. Theoretically, there could be an increased risk for toxicity, especially induced auto-immune effects, due to this quadmodality approach. Indeed, the SBRT boost followed by immunotherapy may prime the immune system to attack not only tumor cells but normal tissue as well.

From a metastatic viewpoint, immunotherapy is an exciting option that is still in its infancy. There are adequate early and non-clinical data suggesting that hypofractionated radiation and immunomodulation may be synergistic. Thus, a more cogent approach to trials addressing both local control and metastatic disease may become “quadmodality” and include combining chemotherapy, conventionally fractionated radiation therapy, immunotherapy and SBRT dose intensification to ablate the residual primary tumor mass. Given the continued devastating effect of lung cancer on the world, such trials need to be developed promptly.

## Author Contributions

Contributors who meet fewer than all four of these criteria for authorship should not be listed as authors, but they should be acknowledged: substantial contributions to the conception or design of the work; or the acquisition, analysis, or interpretation of data for the work; and drafting the work or revising it critically for important intellectual content; and final approval of the version to be published; and agreement to be accountable for all aspects of the work in ensuring that questions related to the accuracy or integrity of any part of the work are appropriately investigated and resolved (SK, KH, and RM).

## Conflict of Interest Statement

The authors declare that the research was conducted in the absence of any commercial or financial relationships that could be construed as a potential conflict of interest.
